# Combined cross-sectional and tangential margin evaluation of different tumor types in dogs and cats

**DOI:** 10.3389/fvets.2025.1629994

**Published:** 2025-10-20

**Authors:** Simona Vincenti, Leonore Aeschlimann, Anna Brunner, Beatriz Vidondo, Vincent Wavreille, Ludmila Bicanova, Sara Soto

**Affiliations:** ^1^Department of Clinical Veterinary Medicine, Vetsuisse Faculty, University of Bern, Bern, Switzerland; ^2^Department of Infectious Diseases and Pathobiology, Vetsuisse Faculty, Institute of Animal Pathology, University of Bern, Bern, Switzerland; ^3^VetSpécialistes, Grand-Saconnex, Switzerland; ^4^Vetsuisse Faculty, Veterinary Public Health Institute, University of Bern, Bern, Switzerland; ^5^AniCura AOI - Animal Oncology and Imaging Center, Hüneberg, Switzerland

**Keywords:** tumors, surgical margins, biopsy trimming, cross sectioning, tangential sectioning, dog

## Abstract

**Introduction:**

Histological evaluation of tumors involves tumor diagnosis and assessment of surgical margins to determine whether they are free (clean) or infiltrated (dirty) by neoplastic cells. In veterinary medicine, cross-sectioning is most commonly used to trim tumors. It is simple, inexpensive, and allows to measure histologic tumor-free distances (HTFD). However, only a minimal portion of the surgical margins are assessed, potentially missing dirty margins. Tangential sectioning evaluates the entire surgical excision border, minimizing the risk of missing dirty margins, but it is more time-consuming, more expensive and HTFD cannot be measured. No study has yet compared these two trimming techniques on different tumors in cats and dogs. Consequently, the main goal of our study was to compare the two trimming techniques and evaluate their agreement.

**Methods:**

We performed both trimming methods and evaluated these parameters in 20 tumors from 13 dogs and 6 cats, on which curative-intent surgical excision was performed. Kappa statistics were calculated to measure agreement between margin evaluation with the two methods.

**Results:**

Cross-sectioning detected dirty margins in 1/20 (5%) tumors. Tangential sectioning identified 11/20 (55%) tumors with dirty surgical margins, including the one detected with the cross-sectioning method (kappa = 0.0826). Ten tumors with dirty margins with the tangential method were not detected as dirty with the cross-sectioning method. Thus, cross sectioning presented a total of 50% false-negative (dirty margins identified as clean margins). The tangential trimming needed a higher number of cassettes and time required for trimming and evaluation.

**Conclusion:**

Based on these results, despite the higher costs, we recommend using a combination of cross and tangential trimming for tumors in cats and dogs.

## 1 Introduction

Histopathologic assessment of tumor surgical margins represents a pivotal stage in tumor management (1–6). Indeed, findings from this evaluation, coupled with clinical presentation and staging, guide clinicians in decision-making regarding further therapeutic interventions (1, 4, 7, 8). The trimming method used to prepare the tumor samples have a significant impact on the histopathological assessment. Several trimming techniques, including cross-sectional (also known as “radial method” or “halves and quarters” method) and tangential sectioning (also called “orange peel” method), have been described in the literature (3, 4, 12). In cross-sectioning, the biopsy is bisected perpendicular to the surgical margin and the lesion, akin to a cross. Owing to its relative simplicity, relative short processing time, and comparatively low costs, this method is most often used on skin tumors in veterinary biopsy services (3, 4, 6, 12). An important advantage of this method is the measurement of histologic tumor-free distances (HTFD), providing valuable clinical information (2, 7, 9, 10). However, it evaluates less than 1% of the overall surgical margin (11, 12), thereby presenting a high risk of false-negative rates. Also, asymmetrically expanding tumors and infiltrative tumors are less suitable for this trimming technique (4, 5, 12). In contrast, the tangential section technique aims for almost a 360° assessment of surgical margins. Here, the excision margins are cut (shaved off) with an orientation parallel to the surgical plane and the entire surface of the surgical margins are then examined for the presence of tumor cells (12). However, tangential trimming precludes HTFD measurement and is notably more time and resource-consuming, resulting in higher costs (4, 6, 12).

A consensus regarding an optimal trimming technique for margin evaluation of tumors in cats and dogs has not been reached, although a combination of cross- and tangential trimming have occasionally been recommended in the literature (2, 4, 6, 10). The main aim of this study is to compare both cross-sectional and tangential trimming methods applied to the same specimen in feline and canine tumors and assess their agreement.

## 2 Materials and methods

### 2.1 Case selection

Twenty primary tumors (*n* = 20) from 13 dogs and 6 cats from the surgical department of the Small Animal Clinic of the Vetsuisse Faculty in Bern were included in the study. The following case information were collected for all animals: age at the time of surgical resection, sex, breed, anatomic location of the mass, biopsy sample, size of the mass, numbers of cassettes used for each technique (cross vs. tangential trimming), time used for each technique (cross vs. tangential trimming), histopathological diagnosis, HTFD, residual tumor classification scheme for each technique (cross vs. tangential trimming) and agreement between triming methods.

### 2.2 Surgery

Upon tumor diagnosis with either FNA and/or incisional biopsy and clinical staging, each patient underwent curative-intent surgical excision with the widest possible surgical margins in relation to tumor anatomical location and expected biological behavior, which was performed by two board-certified small animal surgeons. After surgical resection, landmarks were placed on the biopsies for orientation purposes using sutures (3–0 Nylon Polyamide, Assut sutures, Switzerland) and/or ink (Surgipath Tissue Marking & Margin Dye Kit, Leica Biosystems Nussloch GmbH, Germany). The biopsies were then placed in 10% commercial formalin and submitted for pathological examination to the Institute of Animal Pathology at the Vetsuisse Faculty in Bern.

### 2.3 Pathology

#### 2.3.1 Trimming

Biopsy specimens were fixed in formalin for at least 24 h before sectioning. All cases were macroscopically examined and trimmed by a single experienced board-certified veterinary pathologist. Length, width and depth of the tumor biopsies as well as approximate size of the tumor and location within the tissue were measured and recorded. When available, inks and stitches were also noted and sketched.

**Figure 1:**
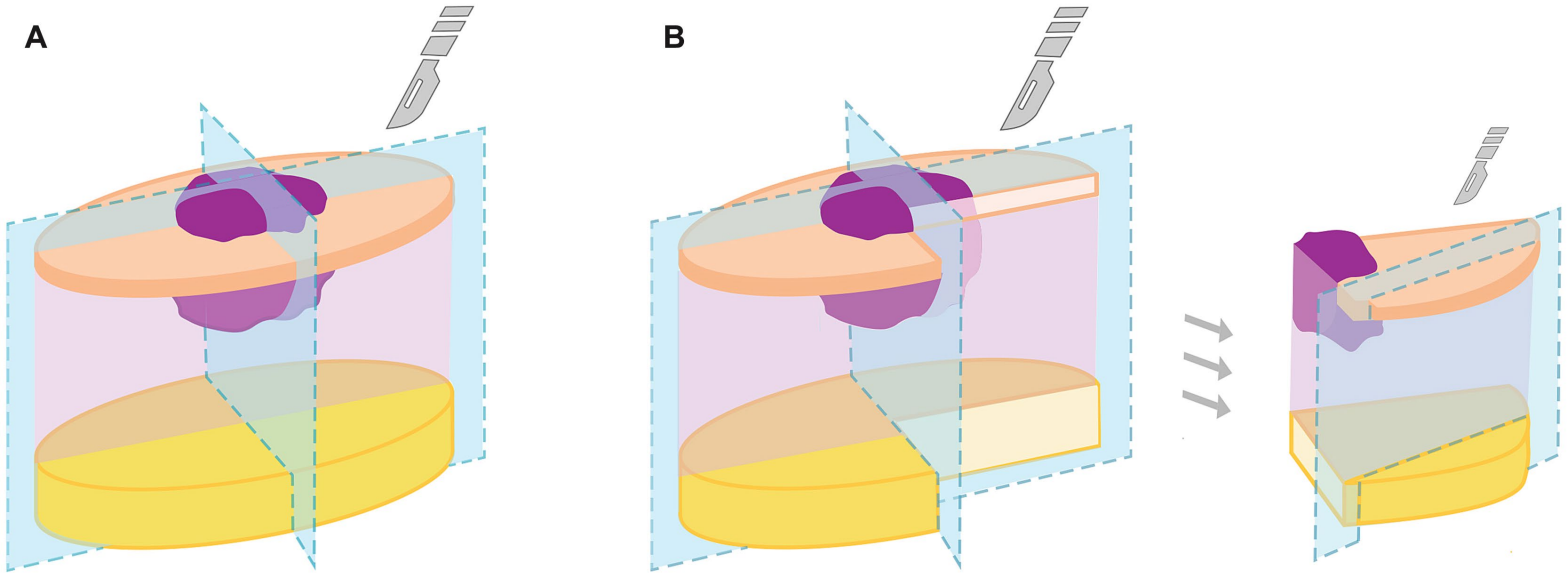
Image depicting how the cross- **(A)** and the tangential trimming **(B)** were performed on the same specimen. **(A)** First, the cross sectioning was performed, then **(B)** the tangential sectioning on each fourth of the specimen. Photo: Moritz Zimmermann.

First, the cross-section (Figure 1A) was performed: The biopsy was trimmed along the shortest axis, including the tumor and the macroscopically narrowest tumor-free surgical margins. Then, an additional section at 90° to the first section was performed, also including the macroscopically narrowest tumor free margins (Figure 1B). All obtained tissue sections had a width of approximately 3–5 mm and were placed in histology cassettes. The number of cassettes used for the cross-sectioning and the estimated time invested were recorded for each case.

Secondly, the margins on either site of the cross-section were cut (shaved-off) tangentially (Figure 1 right). The obtained tissue sections were placed margin-side down in the histology cassettes between two polyester foam pads to ensure that tissue samples were positioned appropriately for paraffin embedding and posterior microtomy. The obtained tissue sections were 3 to 5 mm width. The previously mentioned sketch was again used at this step to note which margin was assigned to which cassette. The number of cassettes used for the tangential sectioning, and the estimated time employed were also recorded.

All tissue sections in the cassettes were processed for routine histopathology, including facing of the paraffin blocks. Facing in this context refers to a step in the production of histology sections. After paraffin embedding, the area to be examined on the block is usually not 100% flat, which is why the surface of the block has to be cut off (smoothed) with the microtome, so that the whole tissue surface appears at the paraffin block surface. The more irregular the surface of the specimen is, the more paraffin and tissue must be removed with the microtome in order to get a complete flat surface of the tissue. After facing, tissue sections of 2–4 μm were cut with the microtome from the paraffin blocks, stained with hematoxylin and eosin (HE), and cover slipped.

#### 2.3.2 Histopathological assessment

All slides were evaluated by two board-certified veterinary pathologists with a Nikon Eclipse Ci-L plus Microscope (Kanagawa, Japan). Tumor diagnosis was assigned for each tumor biopsy based on morphological features. When applicable, a histopathological tumor grading system was used: canine mast cell tumors (MCT) were graded according to the grading schemes of (20, 21) canine soft tissue sarcomas (STS) were graded according to Dennis et al., canine mammary carcinomas were graded according to (22) and feline mammary carcinomas were graded according to Mitotic-Modified Elston and Ellis and Mills et al. methods.

For simplicity, the surgical margins (e.g., lateral, medial, rostral) were divided into two types: (1) deep surgical margin (D), i.e., the surgical excision margin opposite the natural skin surface; and (2) lateral surgical margins (L), i.e., all surgical excision margins except the D margin. The Residual (R) tumor classification scheme, recommended by the American Joint Committee on Cancer and the World Health Organization for human tumors (6, 13) was used to categorize the margins as follows: R0 = complete margin excision (HTFD > 0 mm), R1 = incomplete histologic excision (tumor reaching margin), R2 = intralesional resection with residual grossly evident disease. As residual grossly evident tumor was never observed after surgery, category R2 was not applied in the study. Locations of tumor infiltration (D and/or the L margins) were recorded for R1 cases. For margins free of neoplastic cells, the HTFD of D and L margin was measured. This was defined as the shortest distance of non-neoplastic tissue from the surgical margin to the skin tumor and was measured with a ruler on the histology slides (6, 13). The distances were measured in mm and the smallest margin was defined as <1 mm.

### 2.4 Statistical analysis

The distribution of numerical variables (such us age, number of cassettes and time durations) is presented as mean, median and ranges, while categorical variables (e.g., complete or incomplete margin excision) are presented as counts and proportions. Cohen’s Kappa was calculated to evaluate the agreement between both methods taking into account agreement due to chance. We tested the 1-sided hypothesis that Kappa was larger than zero.

The sample size calculation was carried out based on the results of the first 11 animals. We estimated that a sample size of 20 achieves 81% power to detect a true Kappa value of 0.55 (indicating moderate agreement) at significance level 0.05 (14).

## 3 Results

### 3.1 Case information

Twenty primary tumors (*n* = 20) from 13 dogs and 6 cats from the surgical department of the Small Animal Clinic of the Vetsuisse Faculty in Bern were included in the study (Table 1). One tumor per animal was examined in all cases, except for one dog, for which two tumors in two different anatomical locations were analyzed.

**Table 1:** Histopathological evaluation of the surgical margins of 20 cutaneous tumors in 13 dogs and six cats using the cross-sectioning and the tangential sectioning methods, and classification of the surgical margins following Liptak (19).

Case number	Animal signalment (species, breed, age in years, sex, castration status)	Skin anatomical location	Biopsy sample size† (cm)	Tumor size‡ (cm)	Histo-cassettes used for the cross-sectioning and the tangential sectioning	Time used for cross-sectioning and tangential sectioning (min)	Histopathological diagnosis	Evaluation margins cross-sectioning	HTFD cross-sectioning (mm)	Evaluation margins tangential sectioning	Residual (R) tumor classification scheme		
											Cross-sectioning	Tangential sectioning	Agreement between both trimming methods
1	Dog Mix 6 fc	Left thigh	5.2 × 3.9 × 1.8	2 × 1.9 × 1.5	3 + 15	5 + 30	MCT low grade	Free	D = 3	Free	R0	R0	Yes
									L = 10				
2	Dog German Pinscher 15 fc	4th-5th left mamma	15 × 6 × 6	10 × 6 × 6	5 + 28	5 + 60	Osteosarcoma	Free	D = 1	Infiltrated: D, L	R0	R1	No
									L = 1				
3	Dog Labrador Retriever 12 fc	Dorsal neck	21.5 × 10 × 16	4 × 4 × 1.5	5 + 37	5 + 90	MCT subcutaneous	Free	D = 1	Free	R0	R0	Yes
									L = 20				
4	Dog Lagotto Romagnolo 8 fc	Left elbow	4.1 × 3.8 × 2.4	2.3 × 2.9 × 2.2	4 + 8	5 + 15	STS grade 2	Free	D < 1	Infiltrated: D, L	R0	R1	No
									L = 1				
5	Dog Pekinese 10 fc	4th right mamma	17.4 × 4.8 × 2.2	2.2 × 0.9 × 1.9	4 + 19	5 + 35	Mamma benign mixed tumor	Free	D < 1	Infiltrated: D	R0	R1	No
									L > 20				
6	Dog Rhodesian Ridgeback 10 mc	Left hind limb	4.7 × 4 × 1	1.2 × 0.7 × 0.9	2 + 24	5 + 40	MCT low grade	Free	D = 1	Infiltrated: D, L	R0	R1	No
									L = 9				
7	Dog Mix 12 fc	Left Axilla	6.8 × 4.7 × 4.0	2.7 × 2.0 × 1.8	4 + 15	5 + 30	STS grade 1	Free	D 11	Free	R0	R0	Yes
									L 12				
8	Dog Boxer 5 fnc	Left thigh	4.5 × 6.2 × 2.2	1.2 × 0.6 × 1.4	3 + 14	5 + 30	MCT low grade	Free	D = 6	Free	R0	R0	Yes
									L = 11				
9	Dog Mix 10 mc	Right elbow	9 × 8.7 × 1.2	5.2 × 3 × 0.8	4 + 22	5 + 35	MCT high grade	Free	D = 3	Infiltrated: D	R0	R1	No
									L = 17				
10	Dog Labrador Retriever 13 mc	Left elbow	6.4 × 3.0 × 1.7	0.9 × 0.8 × 1.0	4 + 12	5 + 15	MCT low grade	Free	D = 10	Infiltrated: L	R0	R1	No
									L = 10				
11		Right thigh	3 × 2 × 0.6	0.6 × 0.4 × 0.3	2 + 7	5 + 10	MCT low grade	Free	D = 7	Free	R0	R0	Yes
									L = 5				
	Dog Mops 11 fnc												
12		Left thigh	2.2 × 2.1 × 0.4	0.5 × 0.3 × 0.2	2 + 7	5 + 10	MCT low grade	Free	D = 4	Free	R0	R0	Yes
									L = 7				
13	Dog Lagotto Romagnolo 8 fnc	Chest	7.0 × 5.0 × 2.0	1.7 × 1.0 × 1.7	4 + 14	5 + 15	STS grade 1	Free	D = 6	Free	R0	R0	Yes
14	Dog Mix 12 fnc	1st left Mamma	5.3 × 3.5 × 1.2	1.5 × 1.3 × 0.5	4 + 8	5 + 10	Mamma carcinoma simple	Free	D = 1	Infiltrated: D	R0	R1	No
									L = 8				
15	Cat DSH 14 mc	Between shoulders	11.2 × 9 × 4.1	5.2 × 8.1 × 2.8	5 + 32	5 + 70	Injection site sarcoma	Free	D = 5	Infiltrated: D	R0	R1	No
									L > 25				
16	Cat ESH 12 fc	Right Shoulder	8.8 × 5.3 × 1.7	1.6 × 1.1 × 0.8	5 + 18	10 + 35	Injection site sarcoma	Free	D = <1	Infiltrated: D, L	R0	R1	No
									L = 15				
17	Cat ESH 10 mc	Right lateral neck	6 × 4.1 × 0.6	1.6 × 1.4 × 0.6	4 + 18	5 + 35	Hemangiosarcoma	Free	D < 1	Free	R0	R0	Yes
									L = 7				
18	Cat DSH 12 fc	Right upper lip	2.9 × 2 × 1.4	0.7 × 0.6 × 0.6	2 + 8	10 + 10	SCC	Free	D NA	Free	R0	R0	Yes
									L = 5				
19	Cat Persian 9 fc	Right Mamma	18.7 × 3.3 × 0.9	1.2 × 0.4 × 1.4	3 + 17	5 + 30	Mamma carcinoma	Infiltrated: D	L = 7	Infiltrated: D	R1	R1	Yes
		(mamma complex unknown)											
20	Cat DSH 11 fc	Right medial canthus	1.5 × 0.5 × 0.4	0.5 × 0.5 × 0.2	1 + 3	5 + 10	STS	Free	D NA	Infiltrated: L	R0	R1	No
									L = 1				

DSH, domestic shorthair cat; ESH, European shorthair cat; m, male; f, female; c, castrated; nc, non-castrated; STS, soft tissue sarcoma; MCT, mast cell tumor; SCC, squamous cell carcinoma †, size of the biopsy sample measured macroscopically at the pathology service after formalin fixation; ‡, approximate tumor size measured macroscopically at the pathology service after formalin fixation; HTFD, histologic tumor free distance; D, deep surgical margin, referring to the margin opposite to the skin surface of the tumor biopsy; L, lateral surgical margins; referring to all surgical margins except the deep margin; R0, No tumor cells in the surgical margin (free); R1, Tumor cells in the surgical margin.

Tangential sectioning was more time consuming than cross sectioning due to (1) a larger area that needed to be cut; (2) preparing/labeling of additional cassettes; (3) Sketching of the biopsy to note which tumor margins are placed in which cassette. For the largest tumor, the tangential sectioning took additional 90 min, whereas the smaller tumors took only 10 min to be trimmed tangentially.

Dogs included in this study were 4 mixed breeds, 2 Labrador Retrievers, 2 Lagotto Romagnolos, 1 German Pinscher, 1 Pekinese, 1 Rhodesian Ridgeback, 1 Boxer and 1 Mops, which were between 5 to 15 years old (median 10.2 years). Cats included 3 Domestic Short Hair (DSH) cats, 2 European Short Hair (ESH) cats and 1 Persian cat, which were between 9 to 14 years old (median 11.3 years). *N* = 16 tumors were cutaneous and/or subcutaneous on the limbs (*n* = 8), the neck (*n* = 2), between (*n* = 1) or on (*n* = 1) the shoulder, the axilla (*n* = 1), the chest (*n* = 1), the lip (*n* = 1), and the medial canthus (*n* = 1), and *n* = 4 tumors were located in the mammae.

### 3.2 Trimming and slide manufacturing

After formalin fixation, the resected tumor sizes varied from 1.5 × 0.5 × 0.4 cm to 21.5 × 10 × 16 cm. For cross sectioning, 1 cassette for the smallest and 5 cassettes for the largest biopsies were needed. A mean of 3.5 (median = 4) cassettes were used per case for the cross-sectioning technique. For tangential sectioning, the number of cassettes used varied from 3 for the smallest to 37 for the largest biopsy. On average, 16.5 cassettes (median = 15) per tumor were needed for the tangential technique. The total number of cassettes used for both trimming techniques ranged from 4 to 42, averaging 20 per biopsy (median = 5). Cross sectioning required between 5 to 10 min, whereas for tangential trimming, 10 min for the smallest to 1.5 h for the largest sample were needed. On average, cross sectioning required 5.5 min and tangential sectioning required 30 min.

The paraffin-embedded blocks of the tangential margins required between 400 and 800 μm of facing due to uneven surface (meaning between 400 and 800 μm of the paraffin block surface was cut off before cutting the proper sections). Facing was minimal (<100 μm) for cross sections since their surfaces were nearly smooth.

### 3.3 Histopathological tumor diagnosis

The canine tumors included 8 MCT (1 subcutaneous MCT, 6 low-grade/grade 2 MCT and 1 high-grade/grade3 MCT), 3 STS (2 grade 1 and 1 grade 2), 1 simple mammary carcinoma, 1 mammary benign mixed tumor, and 1 primary mammary osteosarcoma. Tumors of the cats included 3 STS, 2 feline injection site sarcoma (FISS), 1 mammary carcinoma, 1 squamous cell carcinoma (SCC) and 1 subcutaneous hemangiosarcoma.

### 3.4 Histopathological evaluation of cross-sectional and tangential methods

**Figure 2:**
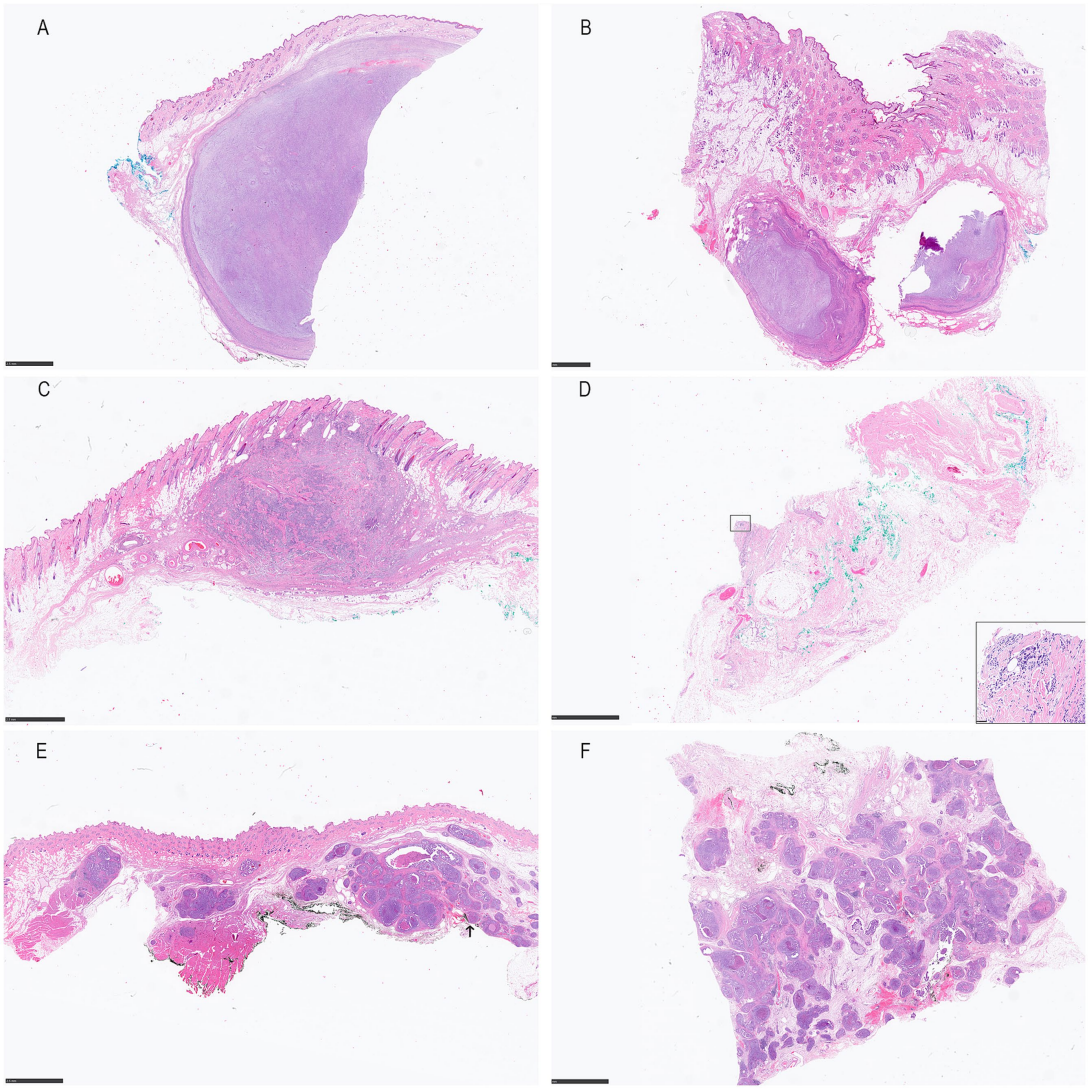
Cross- **(A,C,E)** and tangential- **(B,D,F)** H&E histological sections from a grade II STS in a 8-year old female castrated Lagotto Romagnolo (case 4; **A,B**), a low-grade/grade II MCT in a 10-year old neutered male Rhodesian Ridgeback (case 6; **C,D**) and a tubulopapillary mammary adenocarcinoma in a 9-year old female castrated Persian cat (case 19, **E,F**). Both STS (case 4) and MCT (case 6) present free margins in the cross sections **(A,C)**, while neoplastic cells were found in the tangential sectioning in both cases **(B,D)**. In the feline adenocarcinoma (case 19), neoplastic cells were found at the surgical margin in the cross (figure e, arrow pointing out the dirty deep margin) and in the tangential sections **(F)**.

In 1 skin tumor (5%) (case 19, feline mammary carcinoma), the cross sections revealed presence of neoplastic cells (R1) in the D margin (Figure 2E). The surgical margins of the remaining 19 skin tumor samples (95%) were, when assessed using the cross-sectional technique, free of neoplastic cells (R0) (Figures 2A,C), with variable HTFD.

When assessing the tangential margins, in 11 of 20 (55%) tumor biopsies, including the aforementioned feline mammary carcinoma (Figure 2F), at least one tangential margin was infiltrated by neoplastic cells (R1) (case 2, dog, mammary osteosarcoma; case 4, dog, STS grade 2 (Figure 2B); case 5, dog, mammary benign mixed tumor; case 6, dog, MCT low-grade Kiupel, grade 2 Patnaik (Figure 2D); case 9, dog, MCT high-grade Kiupel, grade 3 Patnaik; case 10, MCT low-grade Kiupel, grade 2 Patnaik; case 14, dog, mammary simple carcinoma; case 15, cat, FISS; case 16, cat, FISS; case 19, cat, mammary carcinoma). In the remaining 9 (45%) tumors, the surgical margins were free of neoplastic cells (R0) by both techniques (case 1, dog, MCT low-grade Kiupel, grade 2 Patnaik; case 3, dog, MCT subcutaneous; case 7, dog, STS grade 1; case 8, dog, MCT low grade Kiupel, grade 2 Patnaik; case 11 and 12, dog, MCT low grade Kiupel, grade 2 Patnaik; case 13, dog, STS grade 1; case 17, cat, hemangiosarcoma; case 18, cat, SCC).

In total, 10/20 (50%) of tumor margins were consistently interpreted as clean (R0) (*n* = 9) or dirty (R1) (*n* = 1) by both methods, while in 10 (50%) cases, cross sectioning resulted in clean margins with variable HTFD, while at least one tangential section was infiltrated by tumor cells.

In the 10 cases in which the assessment of the two techniques did not provide a consistent result, 8/10 (80%) had an HTFD of ≤3 mm at the D or L margin. The remaining two tumors (case 10, dog, MCT low grade Kiupel, grade 2 Patnaik; case 15, cat, FISS) had a HTFD of >3 mm at the affected margin. In the FISS case, the neoplastic cells detected in the tangential technique were present in the D margin. The corresponding cross-section of this tumor had a HTFD at the D margin of 5 mm, including an anatomical barrier. For the canine MCT, the neoplastic cells detected by tangential trimming were observed in a L margin, which in the corresponding cross-section revealed a L HTFD of 10 mm.

Kappa agreement estimate (±95%CI) was very close to zero [0.0826 ± (−0.0774, 0.2426)], showing poor agreement between the cross- and tangential trimming methods.

The histopathological assessment of the tumor margins took on average about 1 min per slide. For cross sectioning, the time invested for the microscopic analysis of the margins varied from 1 min to 5 min per case and took on average 3 min. For tangential trimming, 3 min for the smallest to 37 min for the largest biopsy were needed. On average, tangential sectioning required 16.5 min per case.

## 4 Discussion

Our results show that 1 in 2 (50%) of tumor biopsies will be wrongly interpreted as having complete margin excision (“clean margins”) using the cross-sectional trimming method when they are not. This increase in detection of dirty margins comes, however at a significant increase in both time and costs associated with the tangential trimming method compared to cross-sectioning.

These results are concordant with those previously reported by Dores et al. for canine mast cell tumors^6^, which found that 23.1% tumors with HTFD> 0 mm in the cross sections presented neoplastic cells on the surgical margin on tangential sectioning, a slightly lower value than in our study. In this study, cross sections had 100% specificity for predicting negative tangential margins at a cut-point of 10.9 mm. In contrast to this study, we included different tumors of two different species, and therefore such cut off values for predicting negative tangential margins could not be calculated. In our study, 80% of tumors with R1 margins in the tangential and R0 in the cross section had HTFD of ≤ 3 mm. There were two cases with HTFD >3 mm in the cross section which had R1 margins, namely a canine MCT low-grade/grade 2 and a FISS. The MCT had a HTFD of 10 mm in the cross section, which is below the cut-point indicated by Dores et al. and is consistent with their results. The FISS had a HTFD of 5 mm. Due to the infiltrative and asymmetric growth of this type of tumor (6, 8, 12), it is not surprising that neoplastic cells were present in the tangential margins but not the cross section.

An important aspect that must be considered when assessing tumors histologically is that a certain amount of tissue will always be lost during the manufacturing process of the slides. Tissue blocks need to be trimmed with the microtome to smooth their surface (facing). This loss of tissue will be more pronounced the more irregular the surface of the specimen is. It is therefore crucial to trim biopsies in such a way that the surgical margin can be placed flat into the cassette (15). Placing the tissue sample between two foam pads can minimize the introduction of unnecessary contours and can simplify the subsequent steps in the laboratory. As two perpendicular sections are made in the cross section, the resulting tissue samples usually have a smooth surface, which reduces the need to prepare the surface of the paraffin block. Indeed, less than 100 μm of tissue thickness had to be removed for manufacturing the slides of the cross sections in our study. For tangential sectioning, the surgical margin must be cut around the entire surgical margin, the tissue edges are often irregular. In our study, less than 1 mm (between 400 and 800 μm) of tissue thickness was lost for the tangential margins in the facing step, what in principle should not have major impact on our results. Anyway, it must be taken into consideration that the more tissue cut off during facing, higher are the options of missing neoplastic cells present in this non-assessed tissue area (false clean margins), as well as higher are the options that clean margin tissue may be cut-off, resulting on false dirty margins. Generally, large and irregular samples should be cut into two or more pieces and placed in separate cassettes, to avoid unnecessary tissue loss (4, 15).

In veterinary medicine, many decisions regarding diagnostic tests and treatments are related to cost. Since tumors can be examined easily and with relatively little effort using cross-sectioning, this method became the standard in biopsy services (1, 6, 12), and only rarely the tangential technique is described in the literature for evaluation of tumors (8). To date, the difference in effort and cost between cross-sectioning and tangential trimming has not been thoroughly explored. Our findings reveal a significant increase in time, material and consequently in costs associated with the tangential trimming method compared to cross-sectioning. This difference may discourage many laboratories from adopting the tangential approach. However, given the substantial number of false-negative margins identified with cross-sectioning alone, we stress the importance of incorporating tangential trimming, particularly for certain tumors, such as FISS, where detecting dirty margins can critically impact patient outcomes.

The trimming technique for skin tumors is fundamental to accurate assessment and subsequent post-surgical management. Our study supports the recommendations of Kamstock et al., highlighting that the standard, widely used, and cost-effective cross-sectional method alone could miss up to 50% of dirty margins across various tumor types (4). To address the main limitation of tangential trimming - not giving an indication about the HTFD - we, like (4) advocate for a combined approach. This method integrates cross-sectional and tangential techniques, allowing for the measurement of HTFD while ensuring a thorough evaluation of the margins.

One limitation of our study is the inclusion of two different species and multiple tumor types, though our primary aim was to demonstrate the feasibility of combining both trimming techniques within the same histological sample. Concerning tumor-free margins, as is well known we could not avoid a certain amount of tissue shrinkage which leads to a distortion of the actual dimensions within the living animal (6, 12). Tissue shrinks directly after surgical excision mainly due to muscle contraction and skin elasticity, and due to fixation in formalin. Studies in humans have shown that location and age of the patient influence the degree of this shrinkage (16–18). Histologic measurements, such as HTFD, therefore do not correspond to reality. Neither this, nor a certain amount of tissue loss during the manufacturing process of the slides can be completely avoided. However, careful handling and trimming of the samples as well as measurements before trimming and when evaluating histology slides are important for a thorough assessment of tumor biopsies. Another limitation is the lack of follow-up data. Nonetheless, our main objective was to assess the feasibility and comparative outcomes of applying both cross-sectional and tangential trimming methods to the same specimen. Although the number of cases included in this study is relatively small (20 cases), the results are statistically relevant, as there was a 50% discordance between the two trimming methods: tangential trimming identified margin positivity in 50% of cases that were previously assessed as clean using cross-sectional trimming. Long-term studies are crucial to determine the clinical relevance of these pathological assessments and validate the reliability of these trimming techniques over time. Future research should incorporate follow-up data to provide a more comprehensive understanding of the effectiveness and clinical impact of different trimming methods.

## 5 Conclusion

In conclusion, our study provides strong evidence that the cross-sectional method alone is insufficient for accurate surgical margin evaluation in tumors of cats and dogs. This is especially important for cases in which a revision surgery could be considered (e.g., if the anatomic location allows for another resection or for tumors with notoriously invasive behavior like FISS). Consequently, we recommend that clinicians and pathologists adopt a combined approach, using both cross-sectional and tangential methods, as this will ultimately improve clinical outcomes and guide more effectively our post-operative treatments.

## Data Availability

The original contributions presented in the study are included in the article/supplementary material, further inquiries can be directed to the corresponding author.
